# Bat things come in threes: within-host dynamics of herpesvirus triple infection in bats

**DOI:** 10.1128/jvi.01605-25

**Published:** 2025-10-30

**Authors:** Samantha Aguillon, Magali Turpin, Gildas Le Minter, Camille Lebarbenchon, Axel O. G. Hoarau, Patrick Mavingui, Muriel Dietrich

**Affiliations:** 1 UMR PIMIT (Processus Infectieux en Milieu Insulaire Tropical), Université de la Réunion/INSERM1187/CNRS9192/IRD249 57493, Sainte-Clotilde, France; Cornell University Baker Institute for Animal Health, Ithaca, New York, USA

**Keywords:** Chiroptera, co-infection, herpesvirus, interaction, tropical island

## Abstract

Co-infections are a common feature of wildlife systems, yet the factors influencing within-host viral dynamics remain largely unclear. In bats, understanding viral community ecology is essential for elucidating shedding patterns and potential drivers of zoonotic spillover risk. In this study, we explore the genetic diversity and within-host dynamics of herpesviruses (HSV) in *Mormopterus francoismoutoui*, a tropical insectivorous bat endemic to Reunion Island. Over 3 consecutive years, we collected saliva samples from seven roosts, including samples from recaptured individuals. Illumina sequencing of HSV PCR-positive samples revealed a high diversity of strains (*n* = 20), belonging to alpha, beta, and gamma-HSV subfamilies. Co-infection was frequent, with 44% of bats shedding strains from all three subfamilies. While most shedding patterns appeared random across subfamilies, our results suggest that gamma-HSV negatively affects the probability of alpha-HSV co-shedding. We also demonstrated a lower HSV diversity in juveniles as compared to adults, while pregnancy appeared to increase viral diversity—although this requires further confirmation. Longitudinal recaptures revealed an accumulation of multiple HSV latent infections over life, as the probability to be infected with a new subfamily increased with time interval between recaptures. Within-host strain dynamics were highly variable, with 79% of bats either gaining or losing strains, consistent with latency and reactivation mechanisms. This study provides rare empirical insight into the within-host viral ecology of a natural reservoir host and can help uncover complex pathways by which viruses interact.

**IMPORTANCE:**

Understanding viral dynamics in bats is critical for anticipating and mitigating zoonotic emergence. This study provides rare, longitudinal insight into herpesvirus co-shedding patterns in *Mormopterus francoismoutoui*, an endemic bat species on Reunion Island. Here, we revealed high strain diversity and frequent multi-subfamily shedding, highlighting complex within-host viral ecology, shaped by host age, reproductive status, and infection history. Interactions between viral subfamilies suggest competitive dynamics that may influence shedding. These findings deepen our understanding of viral persistence and reactivation in bats and underscore the need to investigate how environmental and anthropogenic stressors may modulate co-shedding and increase the likelihood of spillover events.

## INTRODUCTION

Multiple infections with distinct virus populations within a single host are common in wildlife (1) and can occur either simultaneously (co-infection) or sequentially (super-infection) (2). Such interactions among co-circulating viruses can modulate viral persistence, replication, and transmission, with implications for host health and pathogen evolution. Within-host interactions may be synergistic, by facilitating genetic exchanges, providing polymerase proteins, and enhancing host immune suppression (3). Conversely, interactions can be antagonistic, by interfering with replication or transmission, via resource competition or interferon-mediated immunity (4, 5). The intensity of within-host competition is expected to be stronger between closely related parasites due, for example, to overlap in resource use and similarity in the elicited immune recognition profiles (2, 6).

Understanding the ecological and functional consequences of within-host viral interactions is particularly important in reservoir species such as bats, which host high diversity of viruses, including many with zoonotic potential. Co-infections are frequent in wild bat populations, with reported prevalence averaging around 42% across multiple studies (7, 8). By altering key epidemiologic factors such as host susceptibility and infection duration (9), co-infections could have important consequences for zoonotic spillovers and disease emergence. Understanding how bat viruses may form interactive communities has thus both significant evolutionary and epidemiological implications (7).

Herpesviruses (HSV) are a valuable model for studying within-host viral interactions due to their high prevalence in diverse host species and their capacity to establish infection shaped by latency phases and reactivation mechanisms (10). HSV family includes three subfamilies with different properties. In humans, alpha-herpesviruses (alpha-HSVs) are known for their rapid lytic replication and latency in non-divided cells (neurons), while beta- and gamma-herpesviruses (beta-HSV and gamma-HSV) establish latency in divided cells and have slower replication cycles, which can lead to persistent or chronic infections, especially in individuals with compromised immune systems (11). Moreover, the lytic cycle is the principal mechanism for alpha- and beta-HSVs, while for gamma-HSV, it is the latency that predominates (11). The ability of HSVs to establish chronic infection, with cycles of reactivation, supports large viral population sizes in the host and can lead to genetic diversification (12). These features make HSVs an ideal model for examining how within-host viral diversity is maintained and shaped over time.

In bats, frequent co-infections have already been reported, with multiple HSV strains and between different subfamilies (beta-HSV and gamma-HSV) (13–16). In contrast, infection with alpha-HSV has only been described in a few bat species and only reported as single infection (17–19). Our recent work on Reunion free-tailed bat suggests that maternally derived antibody protection to HSV in juveniles wanes rapidly and that higher prevalence in male adult bats may be shaped by sex-specific behavior or physiology. Seasonal variation in HSV shedding has also been observed, with peaks occurring during the austral summer (20). However, most existing studies are based on cross-sectional prevalence data and do not assess how host factors and time shape the structure and turnover of within-host viral communities (21–23). In vampire bats (*Desmodus rotundus*) infected with multiple beta-HSV strains, it is suggested that non-competitive strains and latent infections coexist at the population level (21).

In this study, we investigate the genetic diversity and within-host dynamics of HSV in a tropical insectivorous bat, *Mormopterus francoismoutoui*, endemic to Reunion Island. A recent spatio-temporal study revealed that Reunion free-tailed bats are highly infected by HSV (prevalence of 87%, *n* = 3,981 bats) with probable latency mechanisms, explaining long-term viral shedding in saliva (20). In the present study, we cannot distinguish between co- and super-infection. We therefore use the more neutral term co-shedding to describe the co-detection of multiple HSV strains or subfamilies within individual bats. Using a longitudinal design with recaptured individuals over 3 years, we first tested the influence of individual factors (age, sex, and reproductive status) on viral diversity and potential interactions between the co-shedding HSV subfamilies. Within-host dynamics were then assessed, through the recapture of bats, by estimating the probability of changing shedding status (at the subfamily level) and measuring intra-subfamily diversification of HSV strains over time. By linking within-host processes to host traits and viral ecology, our study aims to shed light on the functional dynamics of co-shedding in a natural reservoir host.

## MATERIALS AND METHODS

### Field sampling

Longitudinal monitoring of *M. francoismoutoui* in Reunion Island has been ongoing since 2018 (24). Saliva samples used in this study were collected in 2018, 2019, and 2020 in seven roosts and included data from recaptured bats (totaling between four and five capture events over the 3 years investigated here).

Briefly, bat capture was performed during the dusk emergence as fully described in Aguillon et al. (24), by mainly using harp traps (Faunatech Ausbat) and Japanese mist nets (Ecotone). For each individual, we determined the sex visually and reproductively active status was recorded when females were pregnant and lactating, and in males, when they had large testes (see reference 24 for more details). In females, we recorded the development of nipples as M0 for non-visible nipples, M1 for visible nipples, and M2 for inflated nipples (lactating). Bats were classified as adults or juveniles by examining the epiphysis fusion in finger articulations. Juveniles were identified when articulations were unfused, a characteristic clearly visible up to 7 months of age in this bat species. Beyond this age, some older juveniles may have been mistakenly classified as adults, particularly females without developed nipples (M0). A sterile swab (Puritan Medical Products, USA) was carefully introduced in the corner of the lips to sample saliva and then placed in 250 µL of Eagle minimum essential medium. Samples were stored in a cool box in the field before being transferred the same night at −80°C to the laboratory. Finally, we tattooed bats on the right propatagium with an individual alphanumeric code before releasing them in the capture site. Handling of bats was performed using personal protective equipment, and gloves were disinfected between each individual bat and changed regularly, and all the equipment was disinfected between sites as well (see protocol in Aguillon et al. [24]).

### Molecular analysis and bioinformatics

DNA extraction from saliva samples and amplification of a fragment of HSV DNA polymerase (207 bp product) were mainly performed as part of Aguillon et al.’s study (20). A nested PCR was used to target a broad spectrum of HSVs, including alpha, beta, and gamma subfamilies (25). We took a subset of these data (*n* = 121 PCR-positive samples/3,981 tested bats) and added new samples (processed with the same protocol), to finally include PCR-positive samples from both sexes, from adults and juveniles, from bats in active or non-active reproductive stage, and from bats that have been recaptured several times. This corresponds to three biological periods: putative mating (from April to May, majority of males sampled), pregnancy (from October to December, majority of females sampled), and juvenile weaning (from January to March, majority of juveniles sampled). To prepare samples for Illumina sequencing, the second PCR of the nested protocol was repeated with Illumina adapters. PCR amplicons were then processed following an Illumina MiSeq 250 bp paired-end sequencing method at Macrogen Europe (the Netherlands, Amsterdam), using the Herculase II Fusion DNA polymerase Nextera XT Index V2 library kit.

Raw sequence reads were filtered, cleaned, and trimmed, removing primers and low-quality reads (unexpected length, missing base) using the FROGS 4.0 pipeline (26, 27). Clustering of reads into operational taxonomic units (OTUs) was performed using the SWARM algorithm (28) with an aggregation distance of 5%, followed by the removing of chimeras and the filtering of low proportion OTUs (frequency below 0.005%). Resulting OTUs were considered as putative HSV strains, defined by a 5% nucleotide divergence, although no experimental validation was performed to test whether all OTUs represent biologically distinct strains. Finally, these OTUs were checked in GenBank using the Basic Local Alignment Search Tool (29) to verify their HSV identity. We checked for appropriate sequencing depth per sample by verifying that the percentage of estimated strain diversity covered in each sample ranged between 95% and 100%, using the function *depth.cov* from the R package *hilldiv* (30). To assess the completeness of our sampling, we also created accumulation curves of strain diversity in each roost, with 95% confidence levels based on 1,000 bootstraps, using the R package *iNEXT* (31) (Fig. S1). Strain diversity was measured based on Hill numbers and *q* = 1, which considers both richness and evenness of taxa.

### Phylogenetic analysis

Taxonomic affiliations of HSV strains into the three subfamilies (alpha-, beta-, and gamma-HSVs) were performed using a Bayesian tree in BEAST v.2.6.4 (32). The tree was built using a Yule model and a Hasegawa, Kishino, Yano (HKY) site model with invariant and gamma distribution (33), after model selection using Bayesian information criterion (BIC) with JModelTest v2.1.10 (34). We used a reference data set including sequences previously identified as bat-borne alpha-, beta-, and gamma-HSVs, retrieved from GenBank, ensuring to select a wide range of host bat families and a diversity of strains within each HSV subfamily. Sequence alignment was constructed using ClustalW (35) and MUSCLE (36) and then visually checked in CLC sequence Viewer 7.6.1 (Qiagen Aarhus A/S, Aarhus, Denmark). We used a strict molecular clock with a 100 million chain length and sampling every 10^3^ steps and a burning of 10%. We ran three analyses and combined log outputs (removing 10% of burning for each output) using LogCombiner v2.6.4 (37). Traces of Markov Chain Monte Carlo were checked for convergence of the posterior using Tracer v1.7.1 (38). We combined tree outputs (removing 10% of burning for each output) to obtain a consensus tree using LogCombiner v2.6.4 (37) and then TreeAnnotator v2.6.4 (39) and finally visualized the consensus tree in FigTree v1.4.4 (40).

### Statistical analyses

To assess the effect of age (juveniles vs adults) and sex on HSV strain diversity, we performed generalized linear mixed models (GLMM) including age and sex (and their interaction) as fixed effects and the bat’s ID as a random effect to account for the recapture of some bats. HSV diversity was measured at the strain level, using both the number of strains (modeled as a Poisson distribution, model M1 in Table S1) and Hill numbers (*q* = 1, modeled as a Gaussian distribution with log link, model M2 in Table S1). The analysis of variance function with χ tests was used to test the statistical significance of explanatory variables (and their interactions) by sequentially removing them from the full models. In addition, to assess strain composition differences between both age classes, we performed a permutational multivariate analysis of variance (PERMANOVA) with 10^3^ permutations and visualized results with non-metric multidimensional scaling (NMDS) plot using Bray-Curtis dissimilarity index in FROGS 4.1 (26, 27).

In order to evaluate the effect of reproductive status (active vs non-active) on HSV genetic diversity (Hill numbers, *q* = 1), we used a Gaussian GLMM (with log link) on a subset of data including adult bats during the mating and pregnancy periods. Reproductive status and sex (and their interaction) were included in the model as fixed effects and the bat’s ID as a random effect (model M3 in Table S1). We re-ran the model after excluding non-pregnant females without developed nipples (M0) to avoid including potential misclassified female juveniles in this analysis (model M3bis). Finally, to investigate potential interactions between HSV subfamilies, we used binomial GLMMs on adult bats, modeling the presence/absence of each subfamily using a binomial distribution (see models M4, M5, and M6 in Table S1). Models included reproductive status and sex (and their interaction), as well as the two other subfamilies as fixed effects and the bat’s ID as a random effect (with the exception of model M5 where bat’s ID has been removed because of model failure to converge, and thus, a GLM was used instead).

We investigated within-host dynamics of shedding on a subset of bats (*n* = 11) that have been recaptured (*n* = 45 samples). Specifically, we estimated the probability of acquiring or losing HSV subfamilies through time, by fitting a multinomial logistic regression using the *multinom* function in the *nnet* package, using the R script from Streicker et al. (41). Conversion status was used as a nominal dependent variable with four levels (no changes, gain, loss, gain, and loss), and we defined the non-changing level as baseline outcome against which to compare other shedding status changes (model M7 in Table S1). Finally, we analyzed the evolution of HSV genetic diversity at the individual level, by plotting the intra-subfamily strain diversity (as Hill number *q* = 1) over time for each recaptured bat.

All models were constructed and analyzed in RStudio 1.4.1106 (42) using packages *dplyr*, *effects*, *hilldiv*, *iNEXT*, *lme4*, *ggeffects*, *ggplot2*, and *nnet*.

## RESULTS AND DISCUSSION

### Diversity and abundance of HSV strains and subfamilies

**Fig 1:**
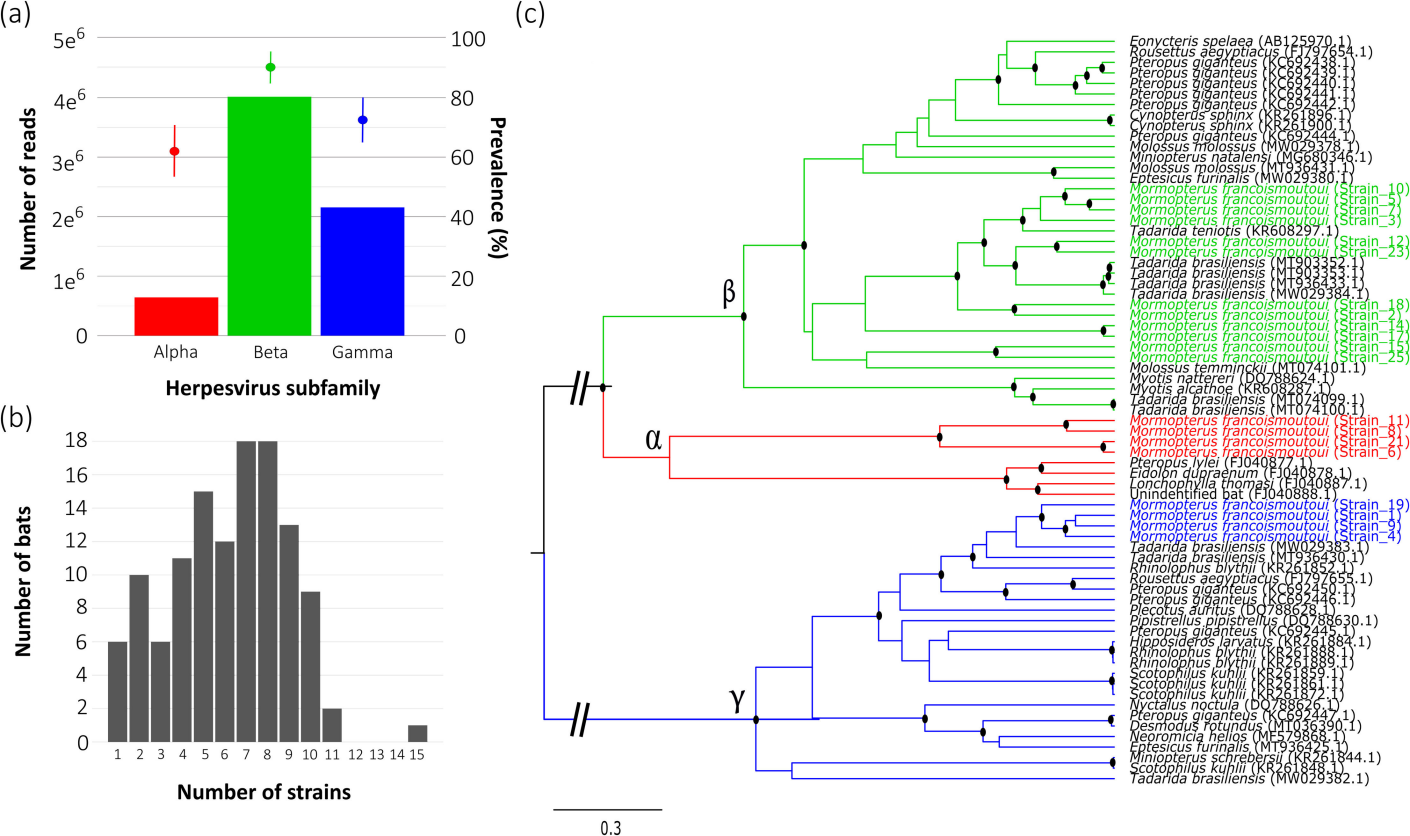
Reunion free-tailed bats are co-infected with HSVs from three subfamilies. (**a**) Abundance of the three HSV subfamilies, based on the total number of Illumina reads over samples. The mean prevalence (proportion of positive samples) detected for each subfamily is indicated by a dot, with a line representing the 95% confidence interval. (**b**) Number of HSV strains hosted by individual bats. (**c**) Phylogeny shows relationships among HSV strains hosted by *M. francoismoutoui* (*n* = 20, colored according to the subfamily) and other bat species (*n* = 63, in black). After alignment, the analyzed fragment of the HSV DNA polymerase was 182 bp long. The analysis was conducted using BEAST with the Yule model and the HKY site model, incorporating invariant sites and a gamma distribution. Nodes with posterior probabilities greater than 0.75 are marked with black dots.

Based on the analysis of 121 saliva samples, we identified a high diversity of HSVs in *M. francoismoutoui*, including 20 strains belonging to the three subfamilies (Fig. 1a). A significant proportion of bats were shedding two (41%) and three (44%) subfamilies. Half of the sampled bats harbored between five and eight distinct strains, with a maximum of 15 strains observed in a single host (Fig. 1b; Fig. S2). These findings confirm that HSV co-infections are common in bats (7). To our knowledge, this study provides the first report of an alpha-HSV in an insectivorous bat (43) and documents the occurrence of triple HSV infection (i.e., co-shedding of three distinct HSV subfamilies) within a single bat species.

Beta-HSV was the most abundant (59% of total reads, Fig. 1a) and diversified (12 strains, Fig. S3) subfamily in *M. francoismoutoui*. Our beta-HSV sequences clustered in several groups closely related to viruses previously found in several molossid bat species, including *Tadarida brasiliensis*, *Tadarida teniotis*, and *Molossus temminckii* (Fig. 1c; 16, 44). Gamma-HSV sequences (32% of total reads, Fig. 1a) grouped in a single cluster closely related to HSV from the mollosid bat species *T. brasiliensis* (16). Alpha-HSV sequences, which were the less abundant (9% of total reads, Fig. 1a), also grouped in a single cluster and were different from those previously found in different nectar-feeding and fruit bat species (no alpha-HSV reference sequences were available for comparison). The close genetic relationships with HSV from other members of the Mollosidae family (at least for beta and gamma subfamilies) illustrate the probable evolution of host specificity in bat HSVs (45, 46). Low abundance of alpha-HSV could be explained by the distinct speed in lytic cycles and mechanisms of reactivation among subfamilies, which may reduce the probability of detecting alpha-HSV in saliva, due to their rapid lytic replication (11). This is coherent with a modeling study suggesting that active phases are much longer than latency phases for beta-HSV dynamics (22), which could explain the higher abundance and diversity of beta-HSV observed in *M. francoismoutoui*.

### Host-associated determinants of HSV strain and subfamily diversity

**Fig 2:**
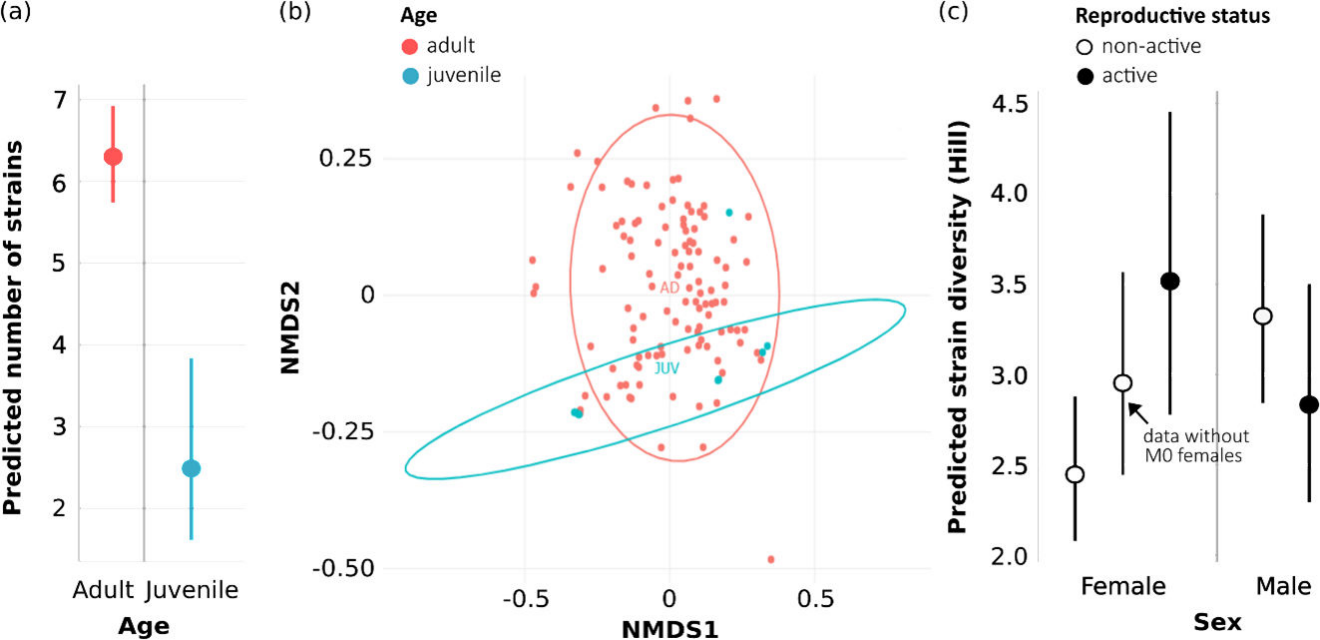
Reduced HSV diversity in juveniles and apparent increased diversity in pregnant females of *M. francoismoutoui*. (**a**) Predicted number of strains according to bat’s age (model M1, Table S1). (**b**) NMDS plot of HSV strain composition according to bat’s age. (**c**) Predicted strain diversity (Hill number, *q* = 1) according to sex and reproductive status of adult bats (model M3, Table S1). For females, strain diversity was also modeled after excluding non-pregnant females without visible nipples (M0) to avoid including potential misclassified female juveniles (model M3bis, Table S1).

Our results reveal strong age-related patterns in within-host HSV diversity. Juvenile bats exhibited significantly lower strain richness and subfamily diversity compared to adults (model M1: χ²_1_ = 20.221, *P* = 7^e−06^, Table S1; model M2: χ²_1_ = 14.118, *P* = 2^e−04^, Table S1) (Fig. 2a; Fig. S4a). This pattern was further confirmed by differences in strain composition (PERMANOVA: *F* = 0.03, *P* < 0.01), with reduced diversity observed in juveniles (Fig. 2b). Indeed, the majority of juveniles (eight of nine) were shedding only one HSV subfamily (beta- or gamma-HSV, Fig. S4b) and a reduced mean number of strains (predicted mean = 2.51, Fig. 2a). Only one juvenile was shedding the three subfamilies (Fig. S4b). In comparison, almost half of adults were shedding the three subfamilies simultaneously (52 over 112, Fig. S4b) and a higher number of strains (predicted mean = 6.44, Fig. 2a). Interestingly, Griffiths et al. (21) did not find an effect of age on the number of HSV strains in *D. rotundus*. This contrasting result could be due to species-specific differences, or because our juvenile class was restricted to newborns up to 4-month-old bats, while Griffiths et al. (21) also included subadults. These subadult bats had probably been exposed to a higher HSV diversity, supported by a previous study on *M. francoismoutoui* from the same population and sampling years (our data set representing a subset), which showed that juveniles get infected rapidly (20). Thus, in addition to reduced HSV prevalence previously reported in juvenile bats (e.g., 13, 47), including in *M. francoismoutoui* (20), our findings revealed a reduced genetic HSV diversity in young juveniles, as compared to adult bats. This supports the hypothesis that maternal antibodies provide early protection, followed by multiple latent HSV infections accumulating over the bat’s lifespan.

In adult bats, we did not detect a global effect of sex on HSV strain diversity (model M3: χ²_1_ = 2.279, *P* = 0.131; Table S1). This is consistent with a recent study on *D. rotundus* (21), although others have shown that sex effect may be variable among bat species (48). However, we observed a positive effect of the reproductive status, dependent on the sex (model M3: χ²_1_ = 9.186, *P* = 0.002; Table S1), as pregnant adult females were shedding a significantly higher HSV diversity compared to the non-pregnant ones (Fig. 2c). A similar pattern has been found in Puerto Rican bats (48), which suggests that reactivation of HSV shedding during pregnancy leads to a diversification of the within-host viral community. However, when excluding non-pregnant females with non-visible nipples (M0), the predicted strain diversity in non-reproductively active adult females increased, and the interaction between sex and reproductive status was no longer observed (model M3bis: χ²_1_ = 3.668, *P* = 0.055; Table S1). This suggests that the pregnancy effect we detected in adult females may be more related to the age of bats, as some non-pregnant M0 females, classified as adults, could in fact be old juveniles (about 11 months old) that were still shedding a lower HSV diversity than true adults.

**Fig 3:**
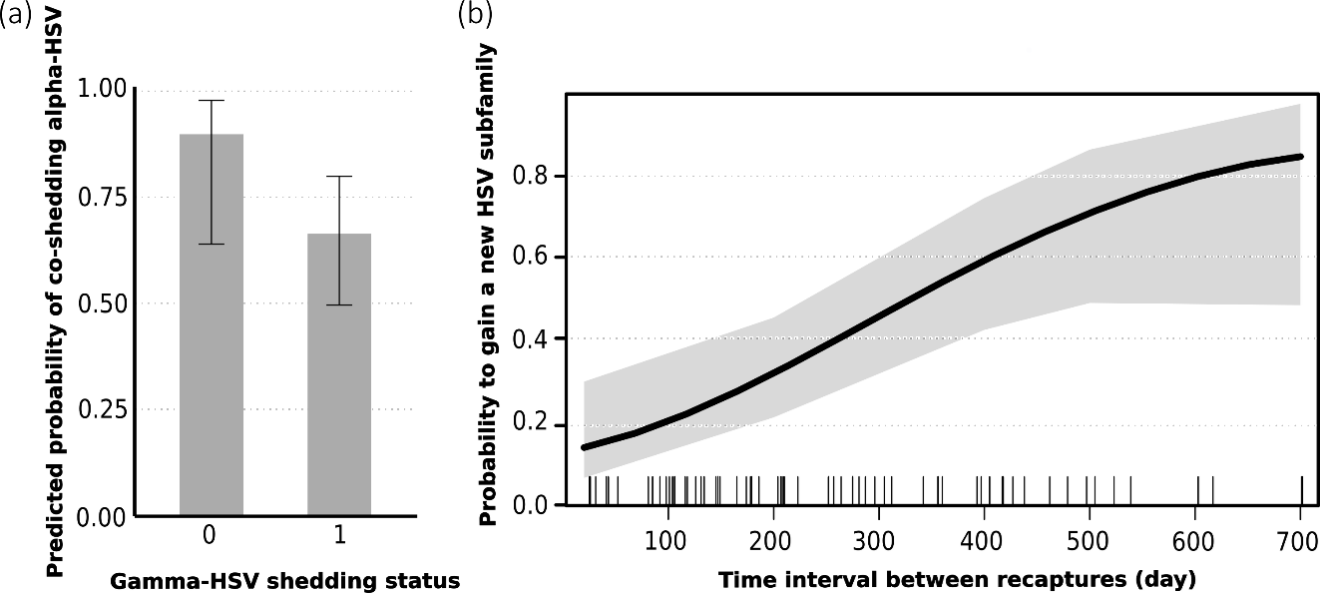
Interaction among HSV subfamilies and their accumulation over life in *M. francoismoutoui*. (**a**) Predicted negative interaction of gamma-HSV on alpha-HSV co-shedding probability (model 4, Table S1). (**b**) Probability to gain a new HSV subfamily over time (model 7, Table S1). Dashes across the *x*-axis indicate recapture of bats, and the shading area represents the 95% confidence interval on the mean probability (solid black line).

Across all sampled bats, alpha-HSV, beta-HSV, and gamma-HSV were detected in 63.6 ± 8.6%, 90.9 ± 5.1 %, and 73.6 ± 7.9% of individuals, respectively. When examining potential interactions among HSV subfamilies, we found no evidence for inhibitory effects of alpha- or gamma-HSV on beta-HSV co-shedding. Indeed, the prevalence of beta-HSV was not influenced by the co-occurrence of alpha-HSV (model M5: χ²_1_ = 3.389, *P* = 0.066; Table S1) and gamma-HSV (model M5: χ²_1_ = 1^e−05^, *P* = 0.997; Table S1). This indicates that patterns of co-shedding between beta-HSV and the two other subfamilies were random, supporting low levels of cross-protective immunity for these pairwise HSV combinations (21). However, adult bats shedding gamma-HSV had less probability to shed alpha-HSV at the same time (model M4: χ_1_ = 4.137, *P* = 0.042; Table S1) (Fig. 3a). Such negative interaction has already been reported in bats but only for HSV strains within the beta-subfamily (49). Further functional investigations on the infection mechanisms of HSV in bats are needed to explore factors leading to these subfamily-specific interaction patterns.

### Within-host dynamics of HSV

Recapture data from 11 adult males over intervals ranging from 25 to 702 days revealed high temporal variability in within-host viral communities. Among them, only two were initially HSV-negative (including one being juvenile at the first capture), and all were then systematically shedding over recapture events. For the majority of individuals (52.86%), bats were shedding the same HSV subfamily over recapture events, although the gain of one or two subfamilies was also largely observed (38.57%). Therefore, we reported few losses or gain/loss events (8.57%). The different categories of transition in HSV shedding status had a different probability to occur over time (model M7: χ²_3_ = 16.06, *P* < 0.001). Indeed, the probability that bats acquire a new HSV subfamily increased over time (Fig. 3b), while the probability to lose or gain/lose was low and stable over time (Fig. S5). These findings are consistent with the biology of HSVs, known for their capacity to establish long-term latency with intermittent reactivation. These results also support that HSV establishes latent infection in bats, as suggested by previous results on prevalence data with bats remaining positive through time (20, 22).

At the HSV subfamily level, investigation of within-host evolution of genetic diversity revealed highly dynamic strain acquisition/release processes (Fig. S6). Indeed, 78.57% of bats both gained and lost strains between captures, and 14.29% of bats only gained strains over time. These frequent shifts are consistent with long-term HSV persistence and may be the result of latency phases and lytic reactivation (21), although it does not exclude the occurrence of clearance processes as well.

### Conclusion

Our work illustrates that HSVs provide an original study system to analyze within-host viral community dynamics in wildlife. The widespread occurrence of triple HSV infections (alpha, beta, and gamma) in *M. francoismoutoui* shed light on individual factors influencing within-host viral diversity and interactions between viruses. Because the precise mechanisms underlying HSV persistence and reactivation remain complex and only partially understood, further functional research is needed. Moreover, HSV reactivation and co-shedding may influence transmission dynamics of other pathogens, including those of zoonotic concern, such as paramyxoviruses and *Leptospira* (20). Future research should thus investigate how environmental or anthropogenic stressors modulate these interactions and explore the evolutionary and epidemiological implications of co-infection in long-lived, gregarious hosts such as bats.

## Data Availability

Sequences have been deposited in NCBI under accession numbers PV034612 to PV034631, and metadata are available in Zenodo (10.5281/zenodo.14810050).

## SUPPLEMENTAL MATERIAL

Supplemental material jvi.01605-25-s0001.docx Table S1; Fig. S1 to S6.

ASM does not own the copyrights to Supplemental Material that may be linked to, or accessed through, an article. The authors have granted ASM a non-exclusive, world-wide license to publish the Supplemental Material files. Please contact the corresponding author directly for reuse.

## References

[1] Hoarau AOG, Mavingui P, Lebarbenchon C. 2020. Coinfections in wildlife: focus on a neglected aspect of infectious disease epidemiology. PLoS Pathog 16:e1008790. doi:10.1371/journal.ppat.1008790 32881983 PMC7470396

[2] Alizon S. 2013. Co-infection and super-infection models in evolutionary epidemiology. Interface Focus 3:20130031. doi:10.1098/rsfs.2013.0031 24516718 PMC3915848

[3] Shartouny JR, Lee C-Y, Delima GK, Lowen AC. 2022. Beneficial effects of cellular coinfection resolve inefficiency in influenza A virus transcription. PLoS Pathog 18:e1010865. doi:10.1371/journal.ppat.1010865 36121893 PMC9521904

[4] Delima GK, Ganti K, Holmes KE, Shartouny JR, Lowen AC. 2023. Influenza A virus coinfection dynamics are shaped by distinct virus-virus interactions within and between cells. PLoS Pathog 19:e1010978. doi:10.1371/journal.ppat.1010978 36862762 PMC10013887

[5] Essaidi-Laziosi M, Geiser J, Huang S, Constant S, Kaiser L, Tapparel C. 2020. Interferon-dependent and respiratory virus-specific interference in dual infections of airway epithelia. Sci Rep 10:10246. doi:10.1038/s41598-020-66748-6 32581261 PMC7314816

[6] Choisy M, de Roode JC. 2010. Mixed infections and the evolution of virulence: effects of resource competition, parasite plasticity, and impaired host immunity. Am Nat 175:E105–E118. doi:10.1086/651587 20297955

[7] Jones BD, Kaufman EJ, Peel AJ. 2023. Viral co-infection in bats: a systematic review. Viruses 15:1860. doi:10.3390/v15091860 37766267 PMC10535902

[8] Wang J, Pan Y, Yang L, Yang W, Lv K, Luo C, Wang J, Kuang G, Wu W, Gou Q, Xin G, Li B, Luo H, Chen S, Shu Y, Guo D, Gao Z-H, Liang G, Li J, Chen Y, Holmes EC, Feng Y, Shi M. 2023. Individual bat virome analysis reveals co-infection and spillover among bats and virus zoonotic potential. Nat Commun 14:4079. doi:10.1038/s41467-023-39835-1 37429936 PMC10333379

[9] Vaumourin E, Vourc’h G, Gasqui P, Vayssier-Taussat M. 2015. The importance of multiparasitism: examining the consequences of co-infections for human and animal health. Parasites Vectors 8:545. doi:10.1186/s13071-015-1167-9 26482351 PMC4617890

[10] Cohen JI. 2020. Herpesvirus latency. J Clin Invest 130:3361–3369. doi:10.1172/JCI136225 32364538 PMC7324166

[11] Weidner-Glunde M, Kruminis-Kaszkiel E, Savanagouder M. 2020. Herpesviral latency-common themes. Pathogens 9:125. doi:10.3390/pathogens9020125 32075270 PMC7167855

[12] Lauring AS. 2020. Within-host viral diversity: a window into viral evolution. Annu Rev Virol 7:63–81. doi:10.1146/annurev-virology-010320-061642 32511081 PMC10150642

[13] Griffiths ME, Bergner LM, Broos A, Meza DK, Filipe A da S, Davison A, Tello C, Becker DJ, Streicker DG. 2020. Epidemiology and biology of a herpesvirus in rabies endemic vampire bat populations. Nat Commun 11:5951. doi:10.1038/s41467-020-19832-4 33230120 PMC7683562

[14] Harima H, Qiu Y, Yamagishi J, Kajihara M, Changula K, Okuya K, Isono M, Yamaguchi T, Ogawa H, Nao N, Sasaki M, Simulundu E, Mweene AS, Sawa H, Ishihara K, Hang’ombe BM, Takada A. 2023. Surveillance, isolation, and genetic characterization of bat herpesviruses in Zambia. Viruses 15:1369. doi:10.3390/v15061369 37376669 PMC10303238

[15] James S, Donato D, de Thoisy B, Lavergne A, Lacoste V. 2020. Novel herpesviruses in neotropical bats and their relationship with other members of the Herpesviridae family. Infect Genet Evol 84:104367. doi:10.1016/j.meegid.2020.104367 32450245 PMC7244429

[16] Moreira Marrero L, Botto Nuñez G, Malta L, Delfraro A, Frabasile S. 2021. Ecological and conservation significance of herpesvirus infection in neotropical bats. Ecohealth 18:123–133. doi:10.1007/s10393-021-01530-2 34184171 PMC8238035

[17] Inagaki T, Yamada S, Fujii H, Yoshikawa T, Shibamura M, Harada S, Fukushi S, Le MQ, Nguyen CT, Nguyen TTT, Nguyen TT, Nguyen TT, Quach VT, Thong VD, Mori K, Sasaki M, Setiyono A, Handharyani E, Takeyama H, Hasebe F, Saijo M. 2020. Characterization of a novel alphaherpesvirus isolated from the fruit bat Pteropus lylei in Vietnam. J Virol 94:e00673-20. doi:10.1128/JVI.00673-20 32669329 PMC7459556

[18] Razafindratsimandresy R, Jeanmaire EM, Counor D, Vasconcelos PF, Sall AA, Reynes J-M. 2009. Partial molecular characterization of alphaherpesviruses isolated from tropical bats. J Gen Virol 90:44–47. doi:10.1099/vir.0.006825-0 19088271

[19] Sasaki M, Setiyono A, Handharyani E, Kobayashi S, Rahmadani I, Taha S, Adiani S, Subangkit M, Nakamura I, Sawa H, Kimura T. 2014. Isolation and characterization of a novel alphaherpesvirus in fruit bats. J Virol 88:9819–9829. doi:10.1128/JVI.01277-14 24942567 PMC4136302

[20] Aguillon S, Turpin M, Le Minter G, Lebarbenchon C, Hoarau AOG, Toty C, Duchet A, Joffrin L, Ramanantsalama RV, Tortosa P, Mavingui P, Dietrich M. 2025. Role of individual and population heterogeneity in shaping dynamics of multi-pathogen shedding in an island endemic bat. PLoS Pathog 21:e1013334. doi:10.1371/journal.ppat.1013334 40644506 PMC12273948

[21] Griffiths ME, Broos A, Bergner LM, Meza DK, Suarez NM, da Silva Filipe A, Tello C, Becker DJ, Streicker DG. 2022. Longitudinal deep sequencing informs vector selection and future deployment strategies for transmissible vaccines. PLoS Biol 20:e3001580. doi:10.1371/journal.pbio.3001580 35439242 PMC9017877

[22] Griffiths M.E, Meza DK, Haydon DT, Streicker DG. 2023. Inferring the disruption of rabies circulation in vampire bat populations using a betaherpesvirus-vectored transmissible vaccine. Proc Natl Acad Sci USA 120:e2216667120. doi:10.1073/pnas.2216667120 36877838 PMC10089182

[23] Sjodin AR, Willig MR, Anthony SJ. 2019. Quantitative delineation of herpesviruses in bats for use in ecological studies. bioRxiv. doi:10.1101/856518

[24] Aguillon S, Le Minter G, Lebarbenchon C, Hoarau AOG, Toty C, Joffrin L, Ramanantsalama RV, Augros S, Tortosa P, Mavingui P, Dietrich M. 2023. A population in perpetual motion: highly dynamic roosting behavior of a tropical island endemic bat. Ecol Evol 13:e9814. doi:10.1002/ece3.9814 36789336 PMC9919472

[25] VanDevanter DR, Warrener P, Bennett L, Schultz ER, Coulter S, Garber RL, Rose TM. 1996. Detection and analysis of diverse herpesviral species by consensus primer PCR. J Clin Microbiol 34:1666–1671. doi:10.1128/JCM.34.7.1666-1671.1996 8784566 PMC229091

[26] Bernard M, Rué O, Mariadassou M, Pascal G. 2021. FROGS: a powerful tool to analyse the diversity of fungi with special management of internal transcribed spacers. Brief Bioinformatics 22. doi:10.1093/bib/bbab318 34410336

[27] Escudié F, Auer L, Bernard M, Mariadassou M, Cauquil L, Vidal K, Maman S, Hernandez-Raquet G, Combes S, Pascal G. 2018. FROGS: find, rapidly, OTUs with galaxy solution. Bioinformatics 34:1287–1294. doi:10.1093/bioinformatics/btx791 29228191

[28] Mahé F, Rognes T, Quince C, de Vargas C, Dunthorn M. 2014. Swarm: robust and fast clustering method for amplicon-based studies. PeerJ 2:e593. doi:10.7717/peerj.593 25276506 PMC4178461

[29] Altschul SF, Gish W, Miller W, Myers EW, Lipman DJ. 1990. Basic local alignment search tool. J Mol Biol 215:403–410. doi:10.1016/S0022-2836(05)80360-2 2231712

[30] Alberdi A, Gilbert MTP. 2019. Hilldiv: an R package for the integral analysis of diversity based on Hill numbers. bioRxiv. doi:10.1101/545665

[31] Hsieh TC, Ma KH, Chao A. 2016. iNEXT: an R package for rarefaction and extrapolation of species diversity (Hill numbers). Methods Ecol Evol 7:1451–1456. doi:10.1111/2041-210X.12613

[32] Bouckaert R, Vaughan TG, Barido-Sottani J, Duchêne S, Fourment M, Gavryushkina A, Heled J, Jones G, Kühnert D, De Maio N, Matschiner M, Mendes FK, Müller NF, Ogilvie HA, du Plessis L, Popinga A, Rambaut A, Rasmussen D, Siveroni I, Suchard MA, Wu C-H, Xie D, Zhang C, Stadler T, Drummond AJ. 2019. BEAST 2.5: an advanced software platform for Bayesian evolutionary analysis. PLoS Comput Biol 15:e1006650. doi:10.1371/journal.pcbi.1006650 30958812 PMC6472827

[33] Hasegawa M, Kishino H, Yano T. 1985. Dating of the human-ape splitting by a molecular clock of mitochondrial DNA. J Mol Evol 22:160–174. doi:10.1007/BF02101694 3934395

[34] Darriba D, Taboada GL, Doallo R, Posada D. 2012. jModelTest 2: more models, new heuristics and parallel computing. Nat Methods 9:772. doi:10.1038/nmeth.2109 PMC4594756 22847109

[35] Thompson JD, Gibson TJ, Higgins DG. 2003. Multiple sequence alignment using ClustalW and ClustalX. Curr Protoc Bioinformatics 00:23. doi:10.1002/0471250953.bi0203s00 18792934

[36] Edgar RC. 2004. MUSCLE: multiple sequence alignment with high accuracy and high throughput. Nucleic Acids Res 32:1792–1797. doi:10.1093/nar/gkh340 15034147 PMC390337

[37] Rambaut A, Drummond AJ. 2015. LogCombiner v1. 8.2. LogCombinerv1 8: 656.

[38] Rambaut A, Drummond AJ, Xie D, Baele G, Suchard MA. 2018. Posterior summarization in Bayesian phylogenetics using tracer 1.7. Syst Biol 67:901–904. doi:10.1093/sysbio/syy032 29718447 PMC6101584

[39] Rambaut A, Drummond AJ. 2019. TreeAnnotator v 2 6.0-MCMC output analysis. Software Development. Part of Beast, 2

[40] Rambaut A. 2018. Figtree software (v. 1.4. 4).

[41] Streicker DG, Fallas González SL, Luconi G, Barrientos RG, Leon B. 2019. Phylodynamics reveals extinction–recolonization dynamics underpin apparently endemic vampire bat rabies in Costa Rica. Proc R Soc B 286:20191527. doi:10.1098/rspb.2019.1527 PMC6790760 31594511

[42] RStudio Team. 2020. RStudio: integrated development for R. Rstudio Team, PBC, Boston, MA.

[43] Gonzalez V, Banerjee A. 2022. Molecular, ecological, and behavioral drivers of the bat-virus relationship. iScience 25:104779. doi:10.1016/j.isci.2022.104779 35875684 PMC9296223

[44] Pozo F, Juste J, Vázquez-Morón S, Aznar-López C, Ibáñez C, Garin I, Aihartza J, Casas I, Tenorio A, Echevarría JE. 2016. Identification of novel betaherpesviruses in iberian bats reveals parallel evolution. PLoS One 11:e0169153. doi:10.1371/journal.pone.0169153 28036408 PMC5201282

[45] Azab W, Dayaram A, Greenwood AD, Osterrieder N. 2018. How host specific are herpesviruses? Lessons from herpesviruses infecting wild and endangered mammals. Annu Rev Virol 5:53–68. doi:10.1146/annurev-virology-092917-043227 30052491

[46] Escalera-Zamudio M, Rojas-Anaya E, Kolokotronis S-O, Taboada B, Loza-Rubio E, Méndez-Ojeda ML, Arias CF, Osterrieder N, Greenwood AD. 2016. Bats, primates, and the evolutionary origins and diversification of mammalian gammaherpesviruses. MBio 7:e01425-16. doi:10.1128/mBio.01425-16 27834200 PMC5101351

[47] Dietrich M, Kearney T, Seamark ECJ, Paweska JT, Markotter W. 2018. Synchronized shift of oral, faecal and urinary microbiotas in bats and natural infection dynamics during seasonal reproduction. R Soc Open Sci 5:180041. doi:10.1098/rsos.180041 29892443 PMC5990816

[48] Sjodin AR, Anthony SJ, Willig MR, Tingley MW. 2020. Accounting for imperfect detection reveals role of host traits in structuring viral diversity of a wild bat community. bioRxiv. doi:10.1101/2020.06.29.178798

[49] Anthony SJ, Epstein JH, Murray KA, Navarrete-Macias I, Zambrana-Torrelio CM, Solovyov A, Ojeda-Flores R, Arrigo NC, Islam A, Ali Khan S, Hosseini P, Bogich TL, Olival KJ, Sanchez-Leon MD, Karesh WB, Goldstein T, Luby SP, Morse SS, Mazet JAK, Daszak P, Lipkin WI. 2013. A strategy to estimate unknown viral diversity in mammals. MBio 4:e00598-13. doi:10.1128/mBio.00598-13 24003179 PMC3760253

