# Supplementary material for: Bat things come in threes: within-host dynamics of herpesvirus triple infection in bats
Source: J Virol. 2025 Oct 30;99(11):e01605-25. doi: 10.1128/jvi.01605-25 (PMC12645971; doi:10.1128/jvi.01605-25)
Supplement: Supplemental material — Table S1; Fig. S1 to S6. [file jvi.01605-25-s0001.docx]

**SUPPLEMENTARY INFORMATION** – *Aguillon et al.*

**Table S1. Summary of the statistical models used to analyse herpesvirus genetic diversity in *M. francoismoutoui*.** Models include all tested variables, with interaction indicated by an asterisk, and significant variables in bold. The percentage of deviance explained by the final model was calculated after comparison to a null model. M0: female with no visible nipples. All the models include Bat’s ID as a random effect (except model M5).

| *Type and model number* | *Analysed samples* | *Response variable* | *Distribution* | *Explanatory variables* | *Xi²* | *p* | *Deviance explained*  *(%)* |
| --- | --- | --- | --- | --- | --- | --- | --- |
| GLMM  M1 | All individuals  n = 121 | Number of strains | Poisson | Age * Sex  Sex  **Age** | 0.038  1.949  **20.221** | 0.846  0.163  **7e-06** | 3.48 |
| GLMM  M2 | All individuals  n = 121 | Hill diversity  (strain level) | Gaussian (log) | Age * Sex  Sex  **Age** | 0.189  1.373  **14.118** | 0.664  0.241  **2e-04** | 4.26 |
| GLMM  M3 | Adults bats  Pregnancy and mating periods  n = 96 | Hill diversity  (strain level) | Gaussian (log) | **Repro * Sex**  Sex  Repro | **9.186**  2.279  7e-04 | **0.002**  0.131  0.979 | 4.74 |
| GLMM  M3bis | Adults bats  Pregnancy and mating periods, without non-pregnant M0  n = 82 | Hill diversity  (strain level) | Gaussian (log) | Repro * Sex  Sex  Repro | 3.668  2e-04  0.150 | 0.055  0.990  0.698 |  |
| GLMM  M4 | Adults bats  Pregnancy and mating periods  n = 96 | Alpha prevalence | Binomial | Repro * Sex  **Gamma**  Beta  Sex  Repro | 0.045  4.137  2.881  1.084  0.152 | 0.833  **0.042**  0.090  0.298  0.696 | 3.88 |
| GLM  M5 | Adults bats  Pregnancy and mating periods  n = 96 | Beta prevalence | Binomial | Repro * Sex  Gamma  Alpha  Sexe  Repro | 5e-09  1e-05  3.389  1.663  3.712 | 0.999  0.997  0.066  0.197  0.054 |  |
| GLMM  M6 | Adults bats  Pregnancy and mating periods  n = 96 | Gamma prevalence | Binomial | Repro * Sex  Beta  Alpha  Sexe  Repro | 1.832  0  0.280  0.020  0.029 | 0.176  1  0.597  0.888  0.865 |  |
| M7 | Recaptured bats  n = 70 (capture events) | Conversion of subfamily | Multinomial | **Time** | 16.06 | 0.001 | 11.78 |


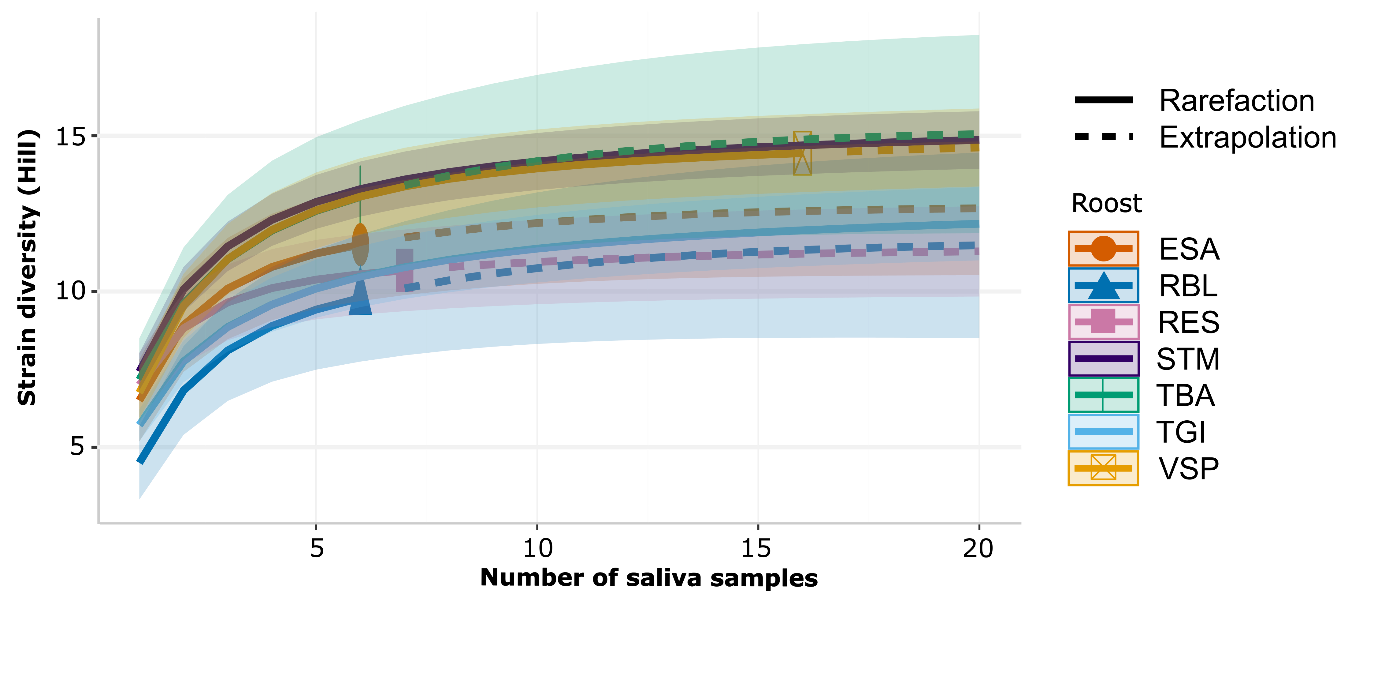


**Figure S1. Accumulation curves of herpesvirus strain diversity (Hill, *q* = 1) according to the number of saliva samples collected per roost.** The curve was constructed based on 1000 bootstraps and the 95% confidence interval is represented by shaded areas.


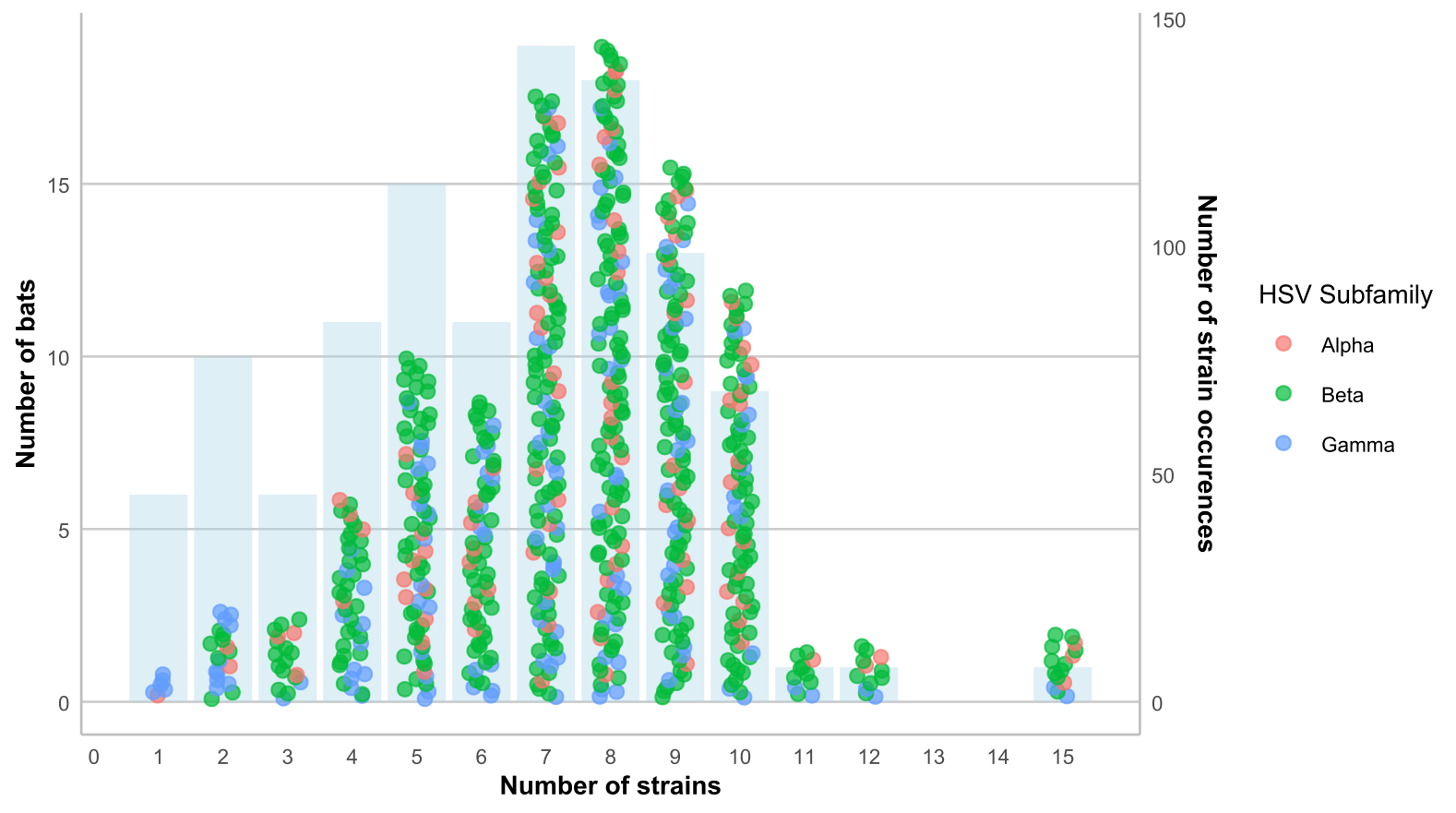


**Figure S2. Distribution of herpesvirus strains in *M. francoismoutoui*.**
The bars indicate the number of herpesvirus strains hosted by individual bats. Within each bar, coloured dots represent the subfamily identity of the detected strains, with one dot per strain. Colours indicate the three HSV subfamilies (alpha in red, beta in green, gamma in blue).

**Figure S3.** **Abundance of the 20 herpesvirus strains identified in *M. francoismoutoui*.** Colors correspond to the three HSV subfamilies (alpha in red, beta in green, gamma in blue). Abundance is determined by the total number of Illumina reads across all samples.


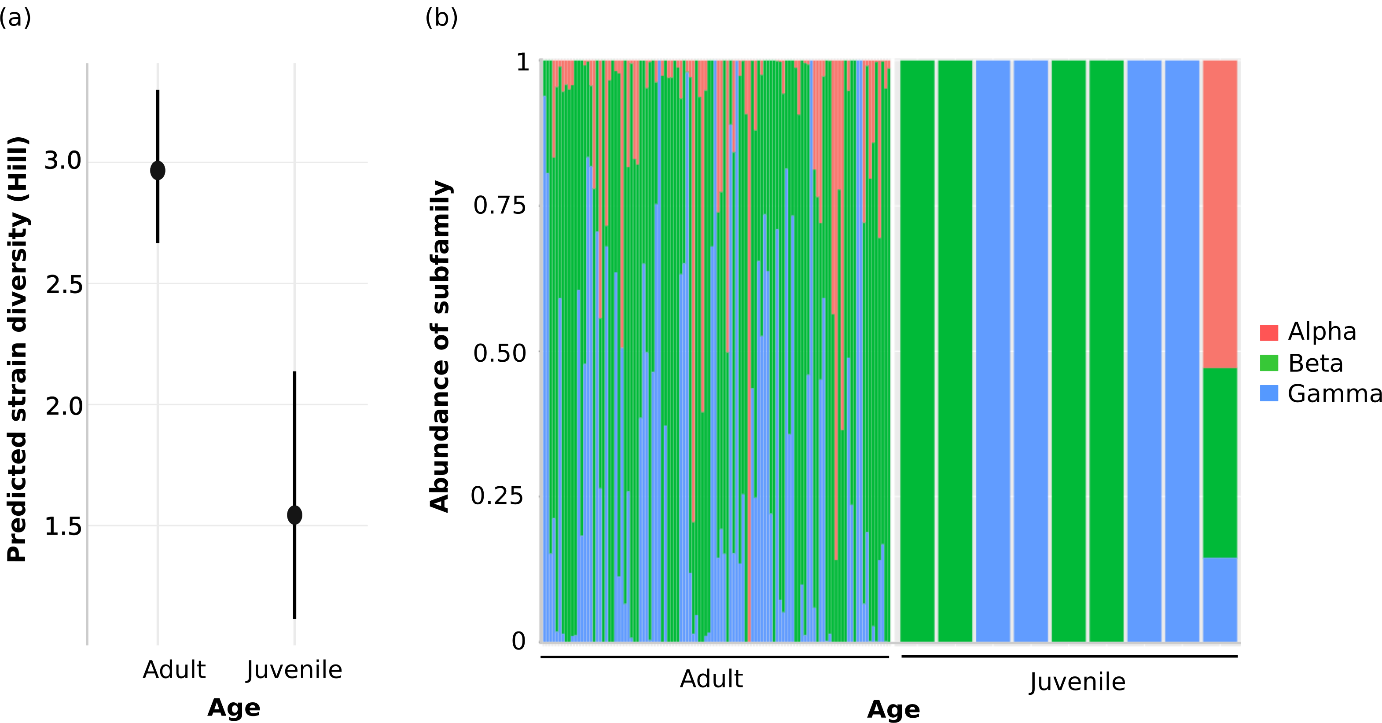


**Figure S4. Diversity of herpesvirus strains and abundance of subfamilies in *M. francoismoutoui* according to bat’s age.** (a) Predicted number of strains in adult and juvenile bats according to model M1 (Table S1). (b) Distribution of herpesvirus subfamilies (color-coded) in adult and juvenile bats, with abundance shown as the relative number of Illumina reads per subfamily for each individual (represented as a column).

**
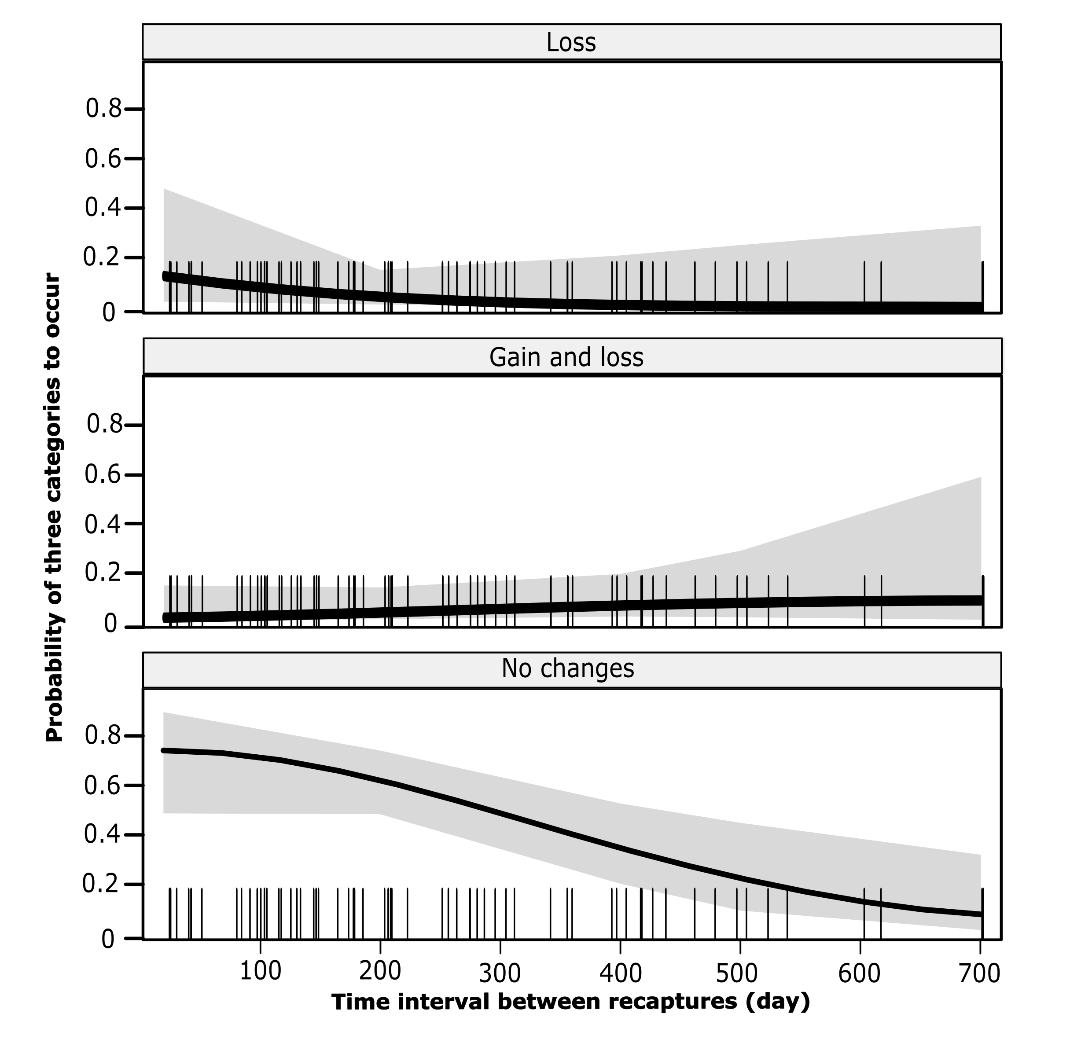
Figure S5. Probability of the three categories of transition in shedding status**. Values were based from predictions from model M7, with 95% confidence intervals in grey. Probability of the fourth category (gain) is presented in Figure 3b.


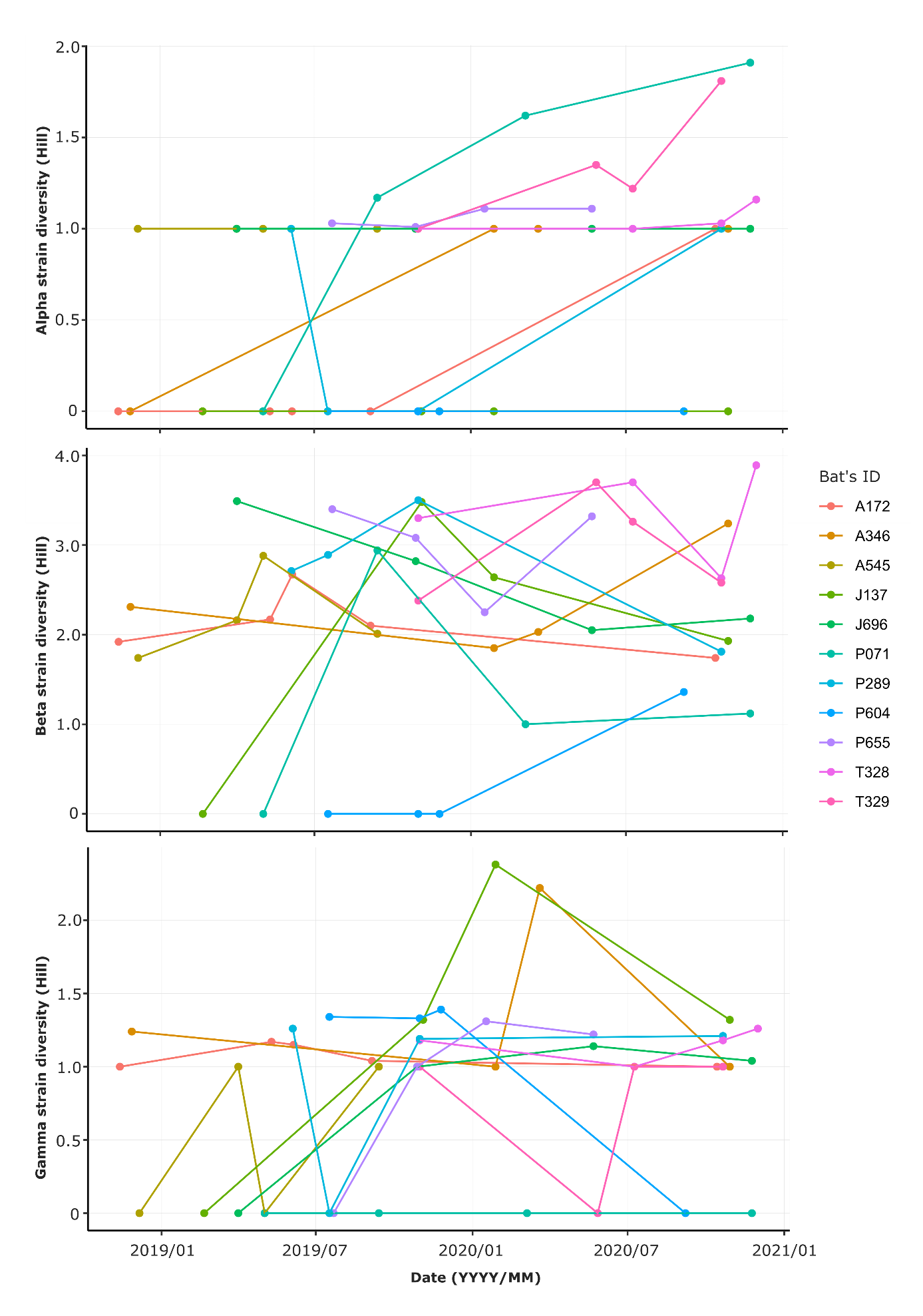
 **Figure S6. Temporal evolution of herpesvirus strain diversity at the individual bat level, for alpha-, beta- and gamma-herpesvirus subfamilies.** Strain diversity was calculated based on Hill numbers (*q* = 1) of herpesvirus strains over time for 11 individual bats captured at least four time.
